# Reversible cardiac disease features in an inducible CUG repeat RNA–expressing mouse model of myotonic dystrophy

**DOI:** 10.1172/jci.insight.143465

**Published:** 2021-03-08

**Authors:** Ashish N. Rao, Hannah M. Campbell, Xiangnan Guan, Tarah A. Word, Xander H.T. Wehrens, Zheng Xia, Thomas A. Cooper

**Affiliations:** 1 Department of Molecular and Cellular Biology,; 2 Department of Molecular Physiology and Biophysics, and; 3 Medical Scientist Training Program, Baylor College of Medicine, Houston, Texas, USA.; 4 Computational Biology Program, Oregon Health & Science University, Portland, Oregon, USA.; 5 Cardiovascular Research Institute, Baylor College of Medicine, Houston, Texas, USA.; 6 Department of Molecular Microbiology and Immunology, Oregon Health & Science University, Portland, Oregon, USA.; 7 Department of Pathology and Immunology, Baylor College of Medicine, Houston, Texas, USA.

**Keywords:** Cardiology, Cell Biology, Arrhythmias, RNA processing

## Abstract

Myotonic dystrophy type 1 (DM1) is caused by a CTG repeat expansion in the *DMPK* gene. Expression of pathogenic expanded CUG repeat (CUGexp) RNA causes multisystemic disease by perturbing the functions of RNA-binding proteins, resulting in expression of fetal protein isoforms in adult tissues. Cardiac involvement affects 50% of individuals with DM1 and causes 25% of disease-related deaths. We developed a transgenic mouse model for tetracycline-inducible and heart-specific expression of human *DMPK* mRNA containing 960 CUG repeats. CUGexp RNA is expressed in atria and ventricles and induced mice exhibit electrophysiological and molecular features of DM1 disease, including cardiac conduction delays, supraventricular arrhythmias, nuclear RNA foci with Muscleblind protein colocalization, and alternative splicing defects. Importantly, these phenotypes were rescued upon loss of CUGexp RNA expression. Transcriptome analysis revealed gene expression and alternative splicing changes in ion transport genes that are associated with inherited cardiac conduction diseases, including a subset of genes involved in calcium handling. Consistent with RNA-Seq results, calcium-handling defects were identified in atrial cardiomyocytes isolated from mice expressing CUGexp RNA. These results identify potential tissue-specific mechanisms contributing to cardiac pathogenesis in DM1 and demonstrate the utility of reversible phenotypes in our model to facilitate development of targeted therapeutic approaches.

## Graphical abstract

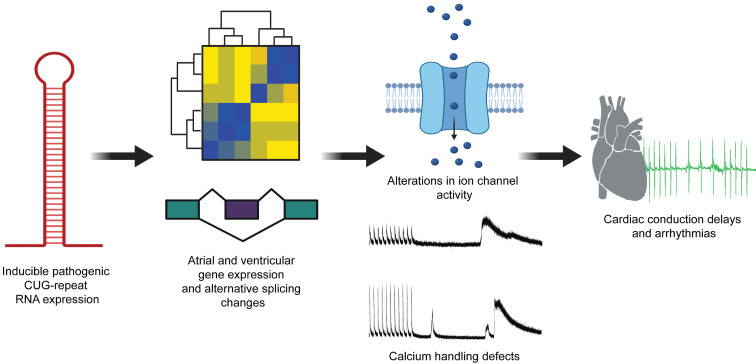

## Introduction

Myotonic dystrophy type 1 (DM1) is the most common form of adult-onset muscular dystrophy characterized by multisystemic manifestations affecting skeletal muscle, cardiac, central nervous system, and gastrointestinal tissues (1). DM1 is an autosomal dominant disease caused by a CTG trinucleotide repeat expansion in the 3′ untranslated region of the *DMPK* gene (2, 3). The gain-of-function expanded CUG repeat (CUGexp) RNA transcribed from the mutated allele is well established as the pathogenic basis for DM1 (4). CUGexp RNA transcripts retained in the nuclei in the form of foci (5, 6) disrupt the activities of the RNA-binding proteins Muscleblind-like (MBNL) (7, 8) and CUGBP, Elav-like family member (CELF) (9, 10), thereby perturbing various aspects of posttranscriptional gene regulation. The best-characterized effects are the disruption of developmentally regulated alternative splicing and polyadenylation programs (11–15).

Cardiac involvement is a prominent feature of DM1 and is the second leading cause of mortality in affected individuals, following respiratory insufficiency due to skeletal muscle wasting (16, 17). In various population studies in patients with adult-onset DM1, cardiac manifestations have been noted in patient groups with a median age of 40 years (18, 19). Recent reports have also described cardiac disease features in children with congenital DM1 (20, 21). The cardiac manifestations in DM1 are multifaceted and primarily consist of conduction delays and both supraventricular and ventricular arrhythmias. Conduction delays include prolongation of the PR interval, QRS complex, and QT interval (22–24). Individuals with DM1 exhibit a variety of arrhythmias, the more common of which are those of supraventricular origin, consisting of sinus node dysfunction, atrial flutter, atrial fibrillation, and supraventricular tachycardia (17, 22, 25). Additionally, affected individuals present with ventricular arrhythmias such as premature ventricular contractions and ventricular tachycardia (24). Histological examination of DM1 heart samples showed fibrosis and fatty infiltration within the conduction system as well as myocyte hypertrophy (26, 27). Additionally, diffuse interstitial fibrosis in the myocardium is also observed (28). Apart from the more common effects on cardiac conduction, a small proportion of affected individuals also exhibit left ventricular hypertrophy, dilatation, and systolic dysfunction (29, 30).

The tissue-specific molecular mechanisms contributing to the complex cardiac phenotypes in DM1 are not well understood. Additionally, there have been a limited number of studies focused on the development of therapeutic strategies for targeting of CUGexp RNA in heart for treatment of cardiac DM1 disease features. Animal models have proven to be an excellent resource for interrogating various aspects of DM1 disease pathology. Mice engineered to model DM1-associated MBNL loss of function by a combined *Mbnl1* and *Mbnl2* knockout (31) or CELF1 gain of function by transgenic overexpression of human CELF1 protein (32) recapitulated several cardiac molecular and physiological disease features. Previously, CUGexp RNA–expressing mouse models of differing repeat lengths have displayed a variable range of DM1-like phenotypes. The CTG_5_ and CTG_200_ models display alternative splicing changes, PR prolongation, and atrioventricular blocks (33, 34). The LC15 model expressing 200 CUG repeats exhibited prolongation of QRS and corrected QT (QTc) intervals and increased susceptibility to ventricular arrhythmias in response to flecainide (class Ic antiarrhythmic agent) administration (35). The EpA960 model (currently unavailable due to extinguished transgene expression) based on Cre-mediated recombination to express 960 interrupted CUG repeats displayed severe cardiac conduction phenotypes and alternative splicing changes in the heart (36). The DMSXL model expressing more than 1000 CUG repeats from a BAC containing an expanded human *DMPK* gene showed weak splicing changes and cardiac conduction abnormalities in aged mice in response to flecainide administration (37).

In this study, we developed a heart-specific mouse model of DM1 based on tetracycline-inducible expression of RNA containing 960 interrupted CUG repeats in the context of human DMPK exons 11–15. Expression of pathogenic CUGexp RNA reproduces many of the cardiac conduction defects and propensity for atrial arrhythmias as observed in individuals affected by DM1. The mice also show MBNL1 and MBNL2 protein colocalization with CUGexp RNA foci and robust splicing defects characteristic of DM1 cardiac tissue. Importantly, the observed electrophysiological, cellular, and molecular phenotypes are reversible upon turning off expression of CUGexp RNA. By RNA-Seq analysis we identified several gene expression and alternative splicing changes in genes associated with the regulation of ion transport in atria and ventricles, which could be associated with phenotypic disease-relevant manifestations. Isolated atrial cardiomyocytes from repeat expressing mice showed calcium-handling defects suggesting a potential mechanism contributing to atrial arrhythmogenesis.

## Results

### Inducible and reversible expression of CUGexp RNA in atrial and ventricular cardiomyocytes.

**Figure 1:**
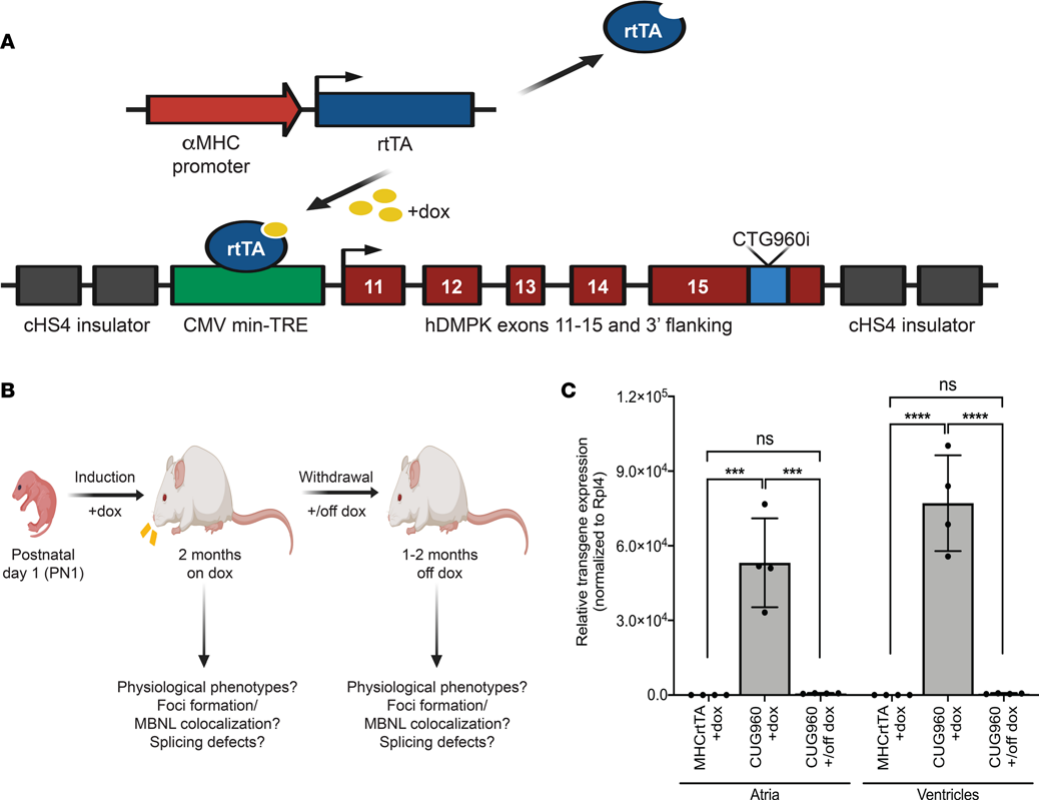
Bitransgenic mouse model for inducible and heart-specific expression of CUGexp RNA. (**A**) The TREDT960I transgene consists of a minimal CMV promoter fused to a tetracycline response element regulating doxycycline induction of RNA containing 960 interrupted CUG repeats in the context of human DMPK exons 11–15. The expression of reverse tetracycline transactivator (rtTA) transgene is driven by a cardiomyocyte-specific α myosin heavy chain promoter. (**B**) Animals were given 2 g/kg dox food for induction of CUG repeat RNA expression beginning at PN1 and characterized for DM1-associated cardiac manifestations. Animals were switched to standard chow to evaluate reversal of disease features in response to cessation of CUGexp RNA expression. (**C**) RT-qPCR analysis of transgene mRNA expression in atria and ventricles of CUG960 mice in response to dox induction since PN1 for 2 months and withdrawal for 2 months in comparison with MHCrtTA +dox control mice. *mRpl4* was used as an internal control for normalization. *n* = 4 animals per group. Data represent the mean ± SD and were analyzed using 1-way ANOVA followed by Tukey’s test for multiple comparisons. ***P < 0.001, ****P < 0.0001. CUGexp, expanded CUG repeat; DM1, myotonic dystrophy type 1; dox, doxycycline; PN1, postnatal day 1.

We developed a mouse model that uses a bitransgenic system for tetracycline-inducible and cardiomyocyte-specific expression of CUGexp RNA. First, we generated transgenic mouse lines bearing the TREDT960I transgene containing 960 interrupted CTG repeats in the context of human DMPK exons 11–15 (38) (Figure 1A). The expression of the transgene is regulated by a minimal CMV promoter fused to a tetracycline response element (TRE). To make the expression of CUGexp RNA heart-specific, TREDT960I mice were crossed with a MHCrtTA line, in which the expression of a modified reverse tetracycline transactivator is regulated by an α myosin heavy chain (αMHC) promoter (39).

Bitransgenic animals used in this study, referred to as CUG960, are homozygous for the TREDT960I transgene and hemizygous for the MHCrtTA transgene. CUG960 animals were given doxycycline-containing (dox-containing) chow to induce expression of CUGexp RNA containing 960 CUG repeats. Mice hemizygous for the MHCrtTA transgene and given dox chow were used as controls for the effects of dox and rtTA expression in cardiomyocytes. CUG960 and MHCrtTA mice were given 2 g/kg dox chow starting on postnatal day 1 (PN1) for a period of 2 months, after which mice were characterized for DM1-associated cardiac phenotypes (Figure 1B). To evaluate reversibility of disease features in response to diminished CUGexp RNA expression, animals were switched to standard chow for either 1 or 2 months and analyzed using the same battery of characterization studies.

Relative expression levels of CUGexp RNA were evaluated by quantitative reverse-transcription PCR (RT-qPCR) analysis in the atria and ventricles of the heart. In comparison with controls, CUG960 +dox mice exhibited a robust induction of transgene expression, which was extinguished in response to dox withdrawal (Figure 1C).

### CUG960 mice displayed physiological phenotypes associated with DM1 cardiac disease.

We evaluated CUG960 +dox mice for the cardiac disease features observed in DM1. Cardiac contractility and morphology were measured by M-mode echocardiography. CUG960 +dox mice displayed no significant differences in ejection fraction, fractional shortening, or intraventricular diameter (Supplemental Figure 1, A–D; supplemental material available online with this article; https://doi.org/10.1172/jci.insight.143465DS1). In comparison with MHCrtTA +dox controls, CUG960 +dox mice exhibited significantly increased left ventricular posterior wall diameter (Supplemental Figure 1, E and F) and larger hearts (approximately 28%) with significantly increased heart weight/tibia length ratios (Supplemental Figure 1G), in agreement with the phenotypes observed previously in a compound muscleblind-knockout model (31).

**Figure 2:**
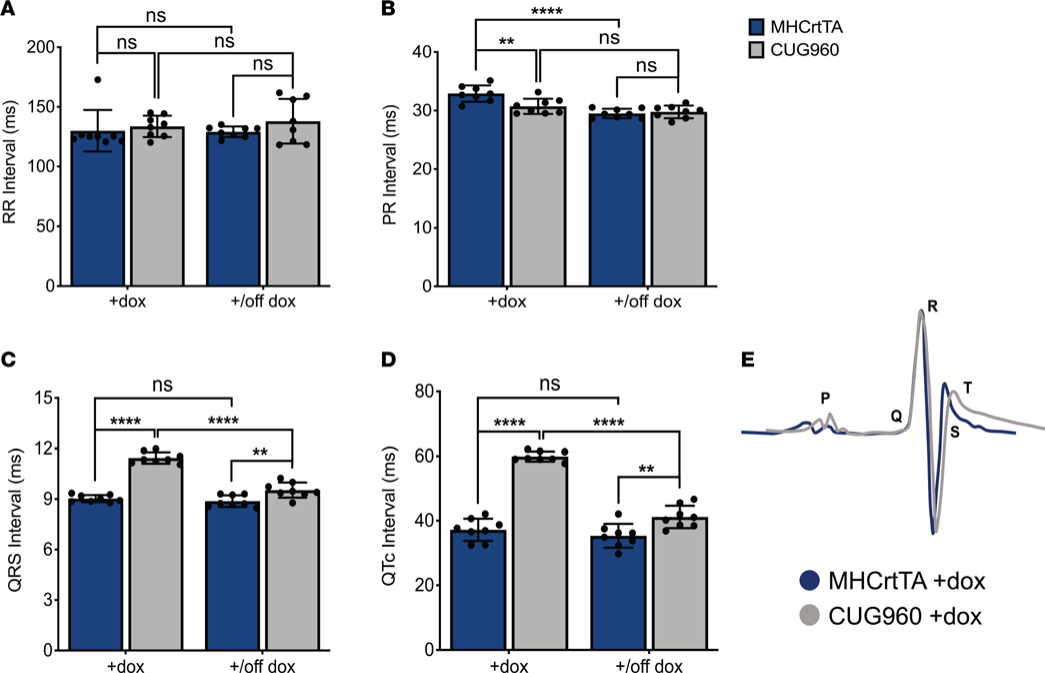
CUG960 mice display reversible conduction abnormalities associated with DM1. Surface ECG recordings were obtained to evaluate conduction intervals in CUG960 and MHCrtTA control mice given dox chow starting at PN1 for 2 months and then switched to regular chow for 2 months: (**A**) RR interval (**B**) PR interval (**C**) QRS interval and (**D**) corrected QT interval (QTc). *n* = 8 animals per group. (**E**) Overlap of representative ECG tracings from MHCrtTA +dox and CUG960 +dox mice. Data represent the mean ± SD and were analyzed using 2-way ANOVA followed by Tukey’s test for multiple comparisons. **P < 0.01, ****P < 0.0001. DM1, myotonic dystrophy type 1; PN1, postnatal day 1; dox, doxycycline.

We examined CUG960 +dox mice for changes in cardiac conduction intervals by surface ECG recordings in anesthetized mice. In comparison with MHCrtTA +dox controls, CUG960 +dox mice did not show prolongation of the RR (Figure 2A) or PR (Figure 2B) intervals, as observed in DM1. However, all CUG960 +dox mice showed robust ventricular conduction defects with prolongation of the QRS (approximately 27% increase) (Figure 2, C and E) and QTc (approximately 61% increase) (Figure 2, D and E) intervals in comparison with control animals. Interestingly, CUG960 mice taken off dox chow for 2 months showed rescue of these parameters in response to diminished CUGexp RNA expression (Figure 2, C and D).

We evaluated whether conduction defects are also observed in animals induced to express CUGexp RNA as adults to exclude any effects of transgene expression on postnatal heart development. CUG960 and MHCrtTA control mice were analyzed by ECG before CUGexp RNA induction at 2 months of age, then after induction on 2 g/kg dox chow for 2 months and then again after a switch to regular chow for 2 months to evaluate reversibility of any observed phenotypes (Supplemental Figure 2A). We observed similar conduction defects — prolongation of QRS and QTc intervals, only on induction of transgene expression, which were rescued following dox withdrawal (Supplemental Figure 2, B and C).

**Figure 3:**
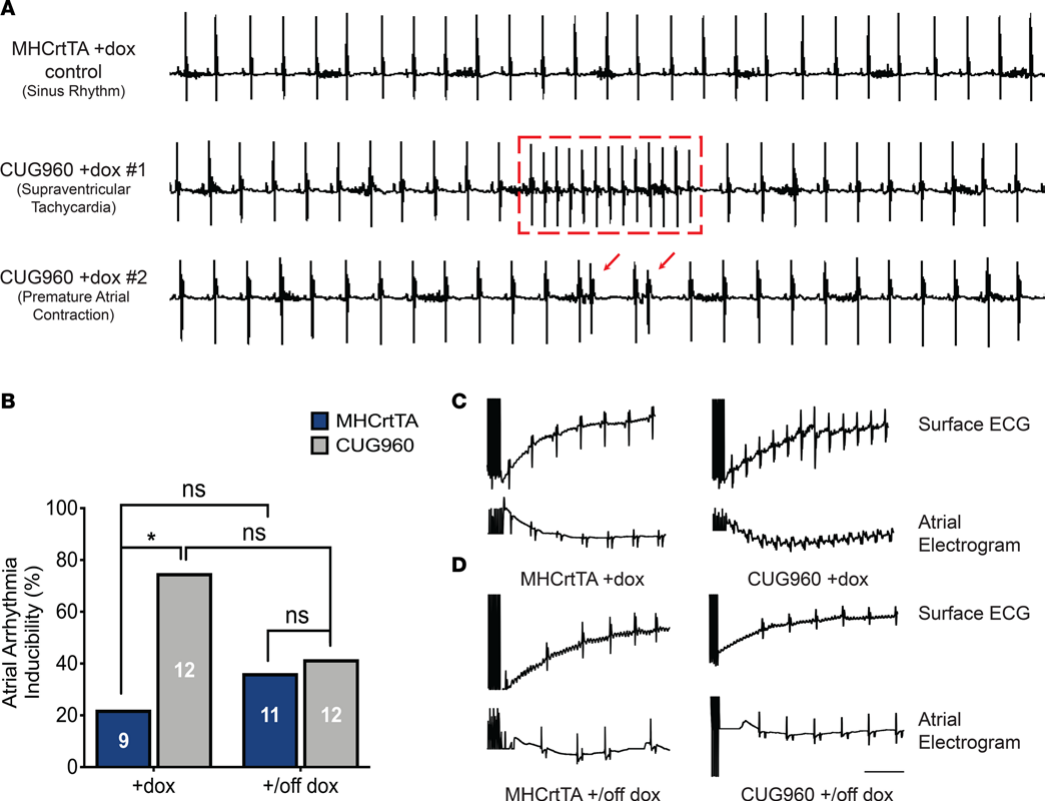
CUG960 mice exhibit an increased and reversible predisposition to atrial arrhythmias. (**A**) Baseline arrhythmias observed in surface ECG recordings for CUG960 +dox mice in comparison with MHCrtTA +dox controls. (**B**) Incidence of atrial arrhythmias induced by electrical pacing in CUG960 vs. MHCrtTA control mice in response to dox induction initiated at PN1 for 2 months and dox withdrawal for 1 month after 2 months of induction initiated at PN1. (**C**) Representative recordings of surface and intracardiac atrial electrograms in CUG960 +dox and MHCrtTA +dox control mice after rapid atrial pacing. (**D**) Representative recordings of surface and intracardiac atrial electrograms in CUG960 +/off dox and MHCrtTA +/off dox control mice after rapid atrial pacing. Numbers of animals analyzed are indicated in corresponding bars. Data were analyzed using Fisher’s exact test. *P < 0.05. Scale bar: 200 milliseconds. PN1, postnatal day 1; dox, doxycycline.

Across multiple cohorts, a proportion of CUG960 +dox mice induced at PN1 (approximately 15%), but none of the MHCrtTA +dox control mice, displayed supraventricular arrhythmias during 1.5–2 minutes of surface ECG recordings (Figure 3A). To evaluate the predisposition of CUG960 +dox mice to atrial arrhythmias in comparison with MHCrtTA +dox controls, we performed intracardiac pacing on 2-month-old CUG960 and MHCrtTA mice, given dox chow beginning at PN1. The incidence of reproducible pacing induced atrial arrhythmias was significantly higher in CUG960 +dox mice (75%, *n* = 12) than in MHCrtTA +dox controls (22.2%, *n* = 9) (Figure 3, B and C). To evaluate reversibility of this phenotype, CUG960 and MHCrtTA animals were taken off dox chow for 1 month after dox induction. In response to diminished CUGexp RNA expression, we observed no significant differences between CUG960 and MHCrtTA animals (Figure 3, B and D), indicating rescue of the increased predisposition to atrial arrhythmias in CUG960 mice.

### CUG960 mice exhibited RNA foci and DM1-associated splicing defects.

**Figure 4:**
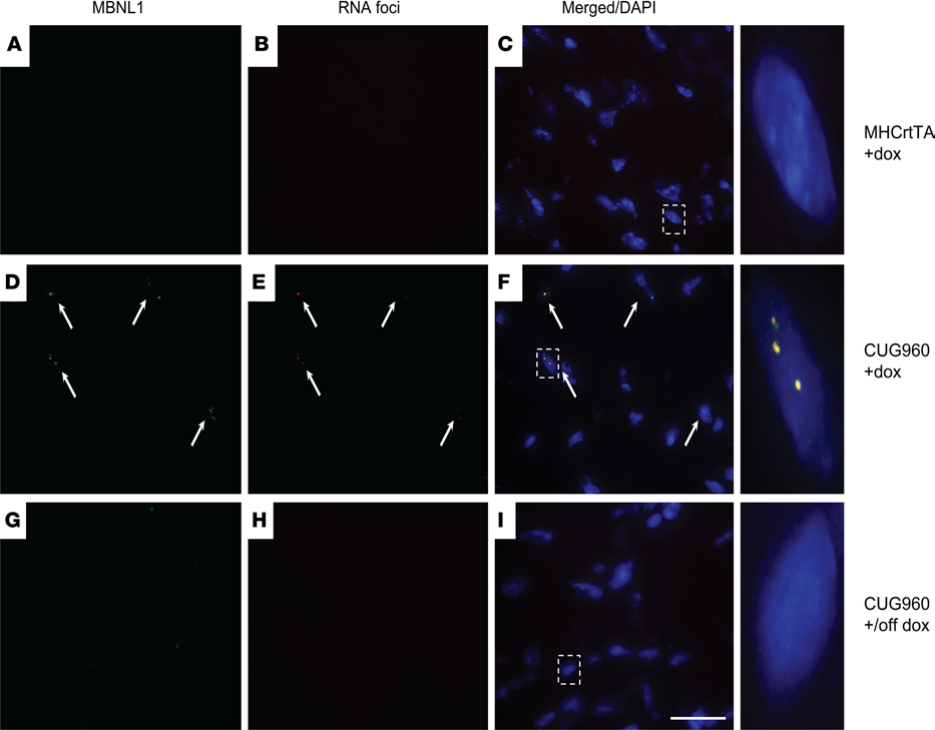
CUG960 mice display reversible nuclear RNA foci formation and MBNL1 colocalization. RNA FISH with a probe targeting CUG repeat RNA combined with immunofluorescence for MBNL1 in the ventricles of CUG960 mice in response to dox induction (**D–F**) and withdrawal (**G–I**) in comparison with MHCrtTA +dox control mice (**A–C**). Arrows indicate RNA foci colocalized with MBNL1 staining. Scale bar: 25 microns. dox, doxycycline.

Aggregation of pathogenic CUGexp RNA into nuclear foci and sequestration of MBNL proteins are key disease hallmarks of DM1, observed in DM1 cardiac tissues (6). To determine whether CUG960 mice display nuclear RNA foci and MBNL colocalization, we performed FISH using Tye-563-labeled (CAG)_5_ locked nucleic acid probes targeting CUGexp RNA combined with IF for MBNL1 or MBNL2. Hearts from CUG960 +dox mice induced to express CUGexp RNA displayed multiple distinct RNA foci in nuclei with colocalization of MBNL1 and MBNL2 proteins (Figure 4, D–F, and Supplemental Figure 3, D–F) and no foci were found to be present in hearts of MHCrtTA +dox control mice (Figure 4, A–C, and Supplemental Figure 3, A–C). CUG960 mice induced with dox chow for 2 months and then switched to regular chow for 2 months (+/off dox) showed no foci formation or MBNL aggregation (Figure 4, G–I, and Supplemental Figure 3, G–I), thereby demonstrating the reversibility of these phenotypes in response to diminished CUGexp RNA expression. Previous studies have implicated elevated CELF1 protein levels in DM1 hearts with disease pathology (10, 40). We evaluated CELF1 protein levels in atria and ventricles of CUG960 +dox and MHCrtTA +dox control mice. In comparison with controls, CUG960 +dox mice did not show a statistically significant change in CELF1 protein levels but variability in the results suggests that some induced animals showed higher levels of CELF1 protein in atria compared with control mice (Supplemental Figure 4, A and B).

**Figure 5:**
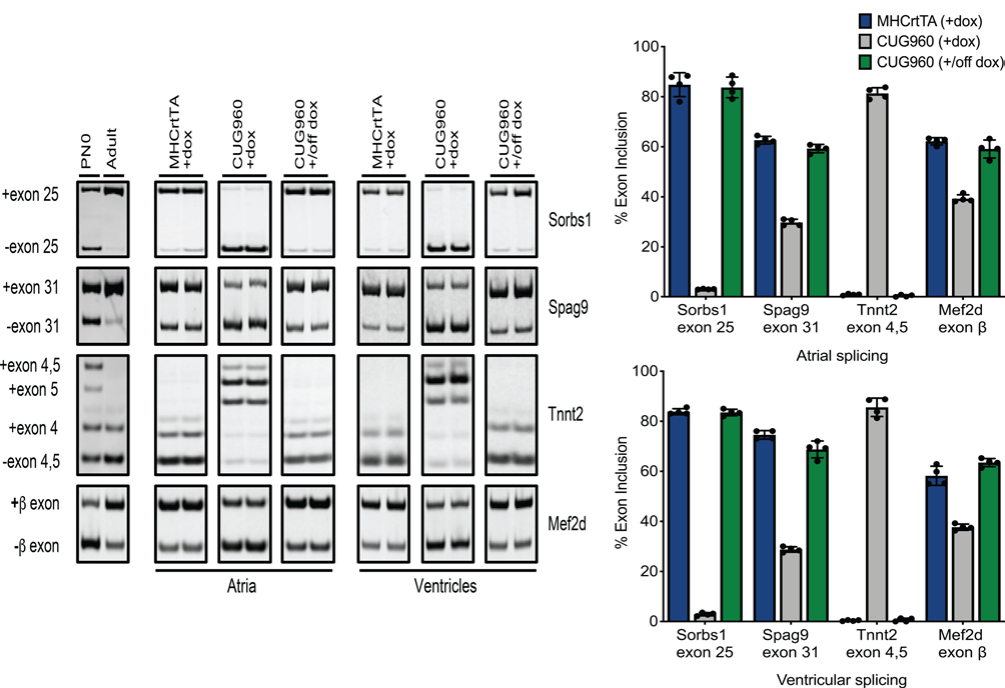
CUG960 mice exhibit strong and reversible DM1-associated splicing defects. Representative RT-PCRs with quantification showing strong reversion to fetal splicing patterns for *Sorbs1* exon 25, *Spag9* exon 31, *Tnnt2* exons 4 and 5, and the *Mef2d* β-exon in atria and ventricles of CUG960 +dox mice in comparison with MHCrtTA +dox controls. Splicing defects are completely rescued in CUG960 mice after dox withdrawal. *n* = 4 animals per group. Data represent the mean ± SD. DM1, myotonic dystrophy type 1; PN0, postnatal day 0 heart; Ad, adult heart ventricle; dox, doxycycline.

A downstream consequence of CUGexp RNA expression is disruption of developmentally regulated alternative splicing with target transcripts showing reversion to fetal isoforms. We evaluated CUG960 +dox mice for splicing events that are (a) conserved in mouse postnatal cardiac development (41) and (b) known to be affected in DM1 individuals (42) and *Mbnl*-knockout mouse models (31, 43). Four splicing events were selected based on these criteria: *Sorbs1* exon 25, *Spag9* exon 31, *Tnnt2* exons 4 and 5, and *Mef2d* β-exon. In comparison with MHCrtTA +dox controls, CUG960 +dox mice showed strong disruption of alternative splicing events in both the atria and ventricles, shifting toward fetal splicing patterns, and these splicing defects could be completely rescued upon turning off transgene expression (Figure 5). To determine the dynamics of the induction and reversibility of splicing defects in response to CUGexp RNA expression, we performed a time course experiment in which adult 6-month-old CUG960 mice were fed dox chow for 3 days, 1 week, or 4 weeks and taken off dox chow for 5 days, 10 days, 16 days, or 4 weeks after 4 weeks of induction (Supplemental Figure 5A). We isolated ventricular RNA from the mice at these time points and evaluated them for the *Sorbs1* exon 25 and *Spag9* exon 31 splicing events. Interestingly, CUG960 +dox mice exhibited a complete splicing switch to the DM1 pattern as early as 3 days on dox chow and nearly complete rescue of these defects by 5 days after dox withdrawal (Supplemental Figure 5B).

### CUGexp RNA expression in the heart led to transcriptomic alterations affecting ion transport genes.

To broadly identify transcriptome changes potentially contributing to the phenotypic manifestations in CUGexp RNA–expressing mice, we performed RNA-Seq analysis on poly(A)-selected RNA from atria and ventricles of 3 CUG960 +dox and 3 MHCrtTA +dox control mice after 10 weeks of induction starting on PN1 (Supplemental Table 1). We obtained an average of 130 million paired-end 150 base pair (bp) reads with 75% or greater reads uniquely mapping to the mouse genome. The quality and depth of the sequencing data were sufficient to identify gene expression and alternative splicing changes associated with CUGexp RNA expression in the heart. For both gene expression (Supplemental Figure 6, A and B) and alternative splicing (Supplemental Figure 6, C and D) data sets, we observed strong correlation between biological replicates indicating high levels of reproducibility.

**Figure 6:**
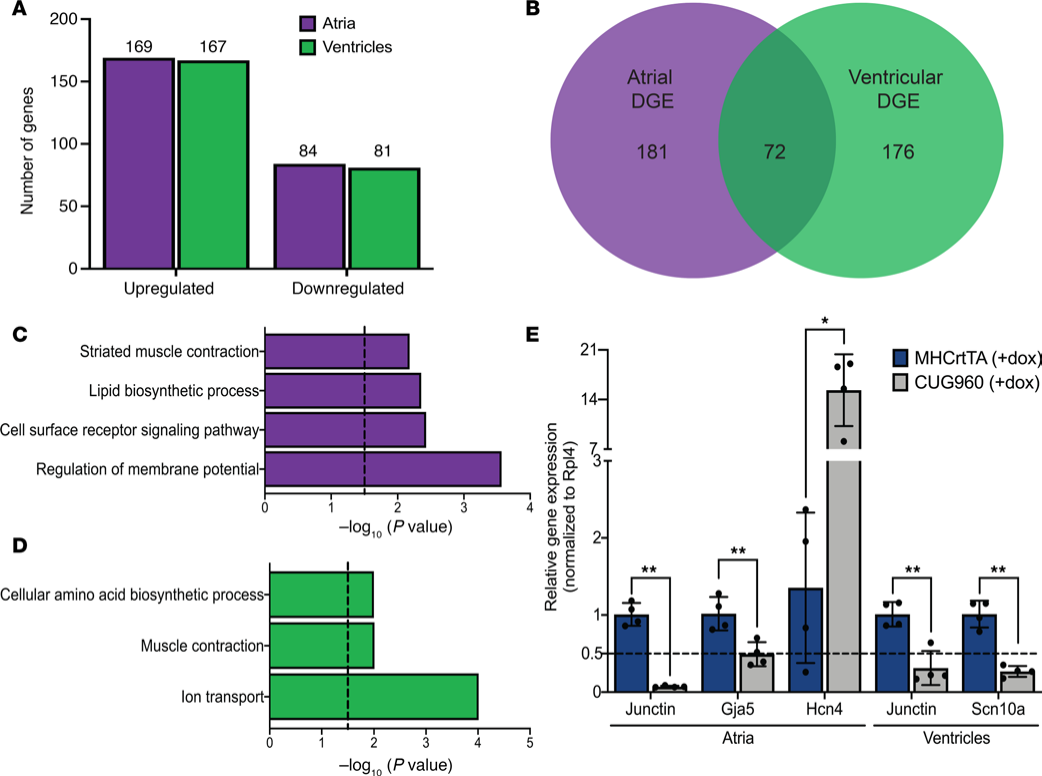
CUGexp RNA induced differential gene expression in atria and ventricles. (**A**) Number of upregulated/downregulated genes in atria and ventricles of CUG960 +dox mice. (**B**) Overlap observed between differentially expressed genes in atria and ventricles of CUG960 +dox mice. Genes showing differential gene expression changes in (**C**) atria and (**D**) ventricles in CUG960 +dox mice were evaluated for enrichment of gene ontology functional terms using DAVID platform. Cutoff > –log (*P* value) = 1.5. (**E**) RT-qPCR-based validation of candidate ion transport genes, *Hcn4*, *Gja5*, *Scn10a*, and *Junctin*, showing gene expression changes in atria and ventricles of CUG960 +dox mice in comparison with MHCrtTA +dox controls. *mRpl4* was used as an internal control for normalization. *n* = 4 animals per group. Data represent the mean ± SD and were analyzed using 2-tailed *t* test. *P < 0.05, **P < 0.01. CUGexp, expanded CUG repeat; dox, doxycycline.

We identified 253 atrial (169 upregulated and 84 downregulated) and 248 ventricular (167 upregulated and 81 downregulated) differentially expressed genes (adjusted *P* value < 0.05, fold change ≥ 1.5) between CUG960 +dox and MHCrtTA +dox control mice (Figure 6A). While genes showing differential expression induced by CUGexp RNA in the atria and ventricles showed less than 30% overlap (Figure 6B), gene ontology analysis revealed enrichment for similar functions of “muscle contraction,” “ion transport,” and “regulation of membrane potential” (Figure 6, C and D). We focused on the category of ion transport genes given their role in regulation of the cardiac action potential and previous associations with cardiac conduction disease (see Discussion). Candidate genes of interest (*Hcn4*, *Gja5*, *Scn10a*, and *Junctin*) with distinct expression patterns in atria or ventricles in CUG960 +dox mice were validated by RT-qPCR analysis (Figure 6E).

**Figure 7:**
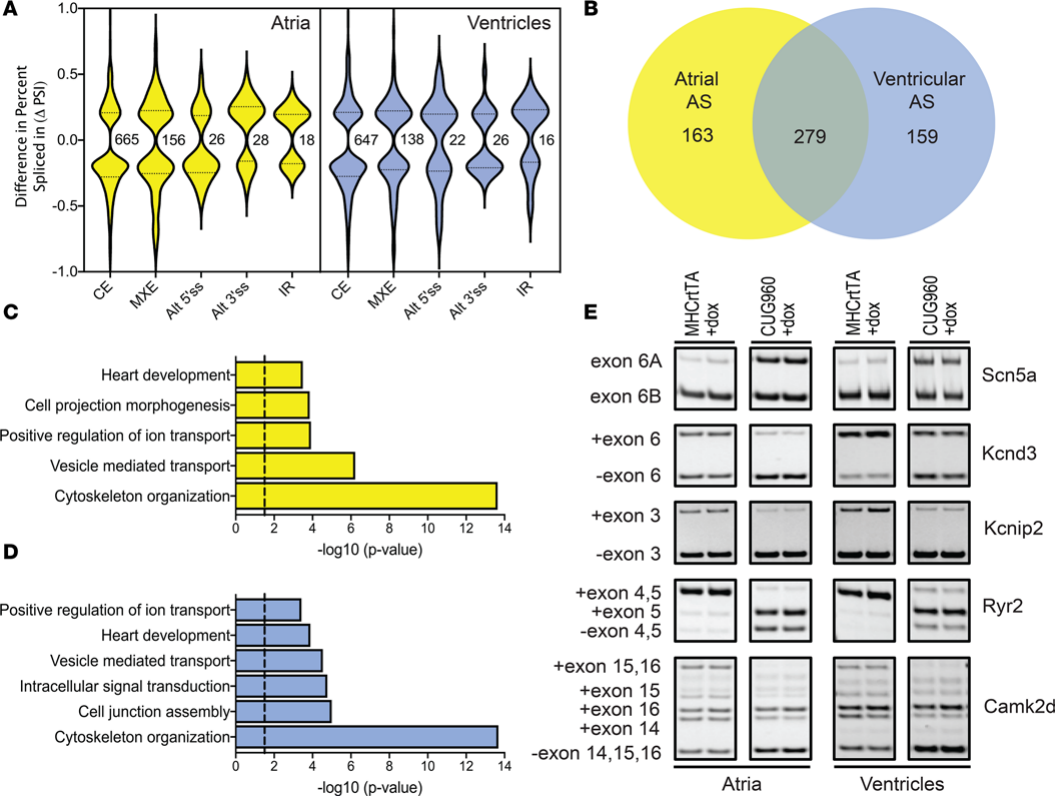
CUGexp RNA induced alternative splicing changes in atria and ventricles. (**A**) Violin plots depicting the distribution of splicing events based on ΔPSI and types of AS changes observed in atria and ventricles of CUG960 +dox mice. (**B**) Overlap observed between genes showing alternative splicing changes in atria and ventricles of CUG960 +dox mice. Genes showing differential alternative splicing changes in (**C**) atria and (**D**) ventricles in CUG960 +dox mice were evaluated for enrichment of gene ontology functional terms using DAVID platform. Cutoff > –log (*P* value) = 1.5. (**E**) Representative RT-PCRs showing alternative splicing changes for candidate ion transport genes, *Scn5a, Kcnd3, Kcnip2, Ryr2*, and *Camk2d*, in atria and ventricles of CUG960 +dox mice in comparison with MHCrtTA +dox controls. *n* = 2 animals per group. CUGexp, expanded CUG repeat; dox, doxycycline; CE, cassette exons; MXE, mutually exclusive exons; Alt 5′ss/Alt 3′ss, alternative 5′/alternative 3’ splice site; IR, intron retention.

In comparison with MHCrtTA +dox controls, we identified 893 events in atria and 849 events in ventricles of CUG960 +dox mice that show differential alternative splicing patterns (FDR < 0.05, ΔPSI ≥ 15%) (Figure 7A). Given the large number of cassette exons affected relative to the other types of alternative splicing events (approximately 75%), we focused on this category for further analysis. In contrast to differential gene expression, we found that genes affected at the level of alternative splicing in atria and ventricles showed primarily similar profiles with approximately 60% overlap (Figure 7B). There was minimal overlap observed between the genes affected at the level of mRNA abundance and alternative splicing in both atria and ventricles (Supplemental Figure 6, E and F), indicating that these groups of genes are affected at distinct nodes of regulation. A total of 3 genes — Necap2 (atria), Coq8a (atria and ventricles), and Pdlim7 (atria and ventricles) — exhibited alternative splicing changes associated with the introduction of a premature stop codon leading to changes in expression levels due to nonsense mediated decay. Gene ontology analysis of genes undergoing changes in alternative splicing in atria and ventricles showed enrichment for common functional terms associated with “cytoskeleton organization,” “vesicle mediated transport,” “positive regulation of ion transport,” and “heart development” (Figure 7, C and D). Similar to our differential gene expression analysis, we focused on alternative splicing events affecting genes involved in regulation of cardiac ion transport and previously implicated in conduction disorders — *Scn5a, Kcnd3, Kcnip2, Ryr2*, and *Camk2d* (Figure 7E). Alternative splicing defects in these genes involved in the regulation of cardiac action potential (*Scn5a, Kcnd3,* and *Kcnip2*) and calcium handling (*Ryr2* and *Camk2d*) lead to production of isoforms with differential properties which could play a pathogenic role in adult tissue (see Discussion).

### Isolated atrial cardiomyocytes from CUG960 mice showed calcium-handling defects consistent with CUGexp RNA-induced transcriptomic alterations.

**Figure 8:**
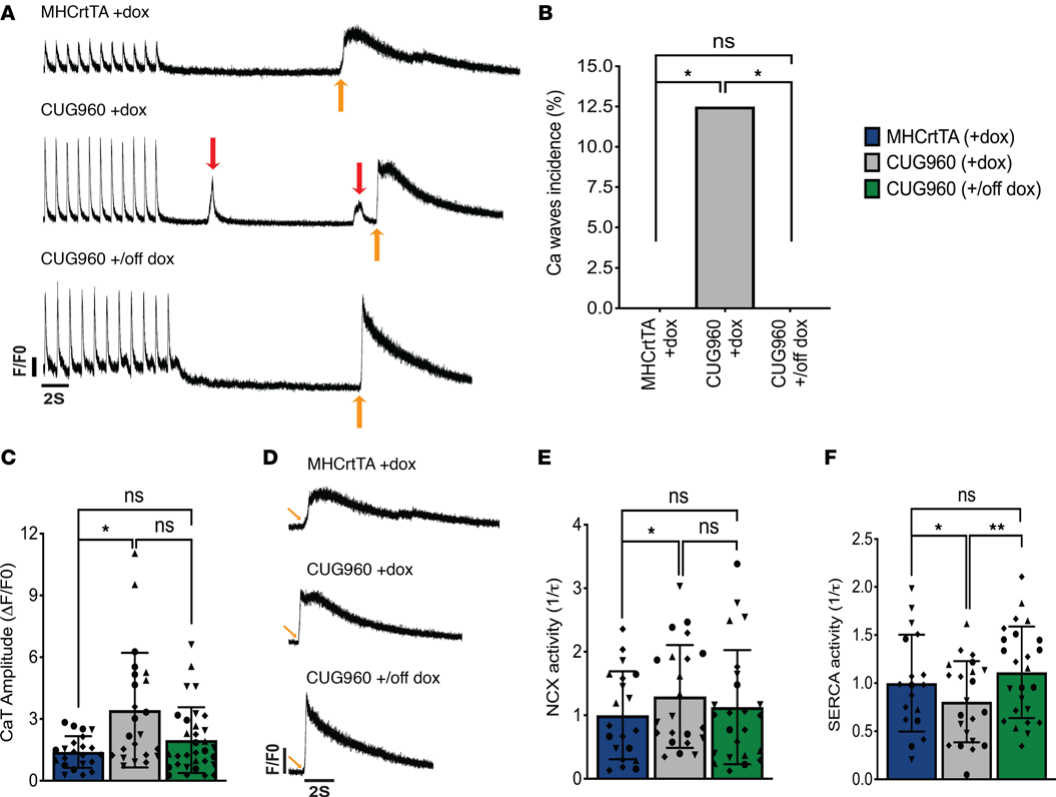
Ca^2+^ handling is altered in isolated atrial cardiomyocytes from CUG960 +dox mice. (**A**) Representative tracings of Ca^2+^ transient recordings during 1Hz pacing and after exposure to 10 mM caffeine, showing spontaneous Ca^2+^ waves in CUG960 +dox mice. Red arrows indicate calcium waves and yellow arrows indicate point of caffeine administration. Traces are drawn to scale with each other. Arbitrary units were used for F/F0. (**B**) Ca^2+^ waves incidence. (**C**) Ca^2+^ transient amplitude. (**D**) Zoomed-in representative caffeine-induced Ca^2+^ transient tracings. (**E**) NCX activity calculated from the decay of the caffeine-induced transient and (**F**) SERCA activity calculated as the difference between the decay of the pacing-induced transient and the caffeine-induced transient in CUG960 +dox mice in comparison with MHCrtTA +dox controls and CUG960 +/off dox mice. *n* = 4 animals per group and data points represent individual cardiomyocytes. Within each group, all cardiomyocytes from each mouse are depicted with distinct symbols. Data represent the mean ± SD. Ca^2+^ waves incidence was analyzed using Kruskal-Wallis 1-way ANOVA followed by Dunn’s test for multiple comparisons. All other data were analyzed using the Generalized Estimating Equation function in SPSS. *P < 0.05, **P < 0.01. CaT, Ca^2+^ transient; NCX, Na^+^/Ca^2+^ exchanger; SERCA, sarco/endoplasmic reticulum calcium ATPase; dox, doxycycline.

Defects in calcium handling are significant contributors to the pathophysiology of atrial arrhythmias (44). Given the increased predisposition to atrial arrhythmias and expression or alternative splicing differences in calcium-handling genes observed in CUGexp RNA–expressing mice, we performed calcium imaging studies in atrial cardiomyocytes isolated from CUG960 +dox mice in comparison with MHCrtTA +dox controls to probe for perturbations in calcium cycling. In comparison with controls, CUG960 +dox mice did not show differences in spontaneous calcium spark frequency or sarcoplasmic reticulum (SR) load (Supplemental Figure 7, A–C). However, CUG960 +dox mice exhibited a higher incidence of diastolic spontaneous calcium waves (Figure 8, A and B) and increased systolic calcium transient amplitude (Figure 8C), suggestive of increased RyR2 activity. Additionally, myocytes demonstrated increased Na^+^/Ca^2+^ exchanger (NCX) (Figure 8, D and E) and decreased sarcoendoplasmic reticulum calcium ATPase (SERCA) current (Figure 8F). In response to diminished CUGexp RNA expression, CUG960 mice displayed rescue of increased calcium waves incidence (Figure 8, A and B) and decreased SERCA current (Figure 8F).

## Discussion

Despite 50% of patients with DM1 having cardiac manifestations, the tissue-specific mechanisms that contribute to cardiac disease features are not well understood. To model DM1-associated RNA toxicity in the heart, we developed transgenic mice for inducible and cardiomyocyte-specific expression of 960 CUG repeats in the natural context within human *DMPK* exon 15. In comparison with controls, CUG960 +dox mice exhibited DM1-associated prolongation of QRS and QTc conduction intervals. CUG960 +dox mice showed spontaneous supraventricular arrhythmias and a higher predisposition to pacing-induced atrial arrhythmias. CUG960 +dox mice also showed the presence of nuclear foci, colocalization of MBNL1 and MBNL2 proteins with the foci, and strong reversion to fetal alternative splicing patterns in both the atria and the ventricles. The cardiac conduction abnormalities, RNA foci formation, MBNL colocalization and alternative splicing defects were rescued in response to diminished CUGexp RNA expression.

Transcriptomic analysis of atrial and ventricular RNA isolated from CUG960 +dox mice revealed differential expression and alternative splicing changes in several genes involved in the regulation of cardiac ion transport (Figure 6 and Figure 7). CUG960 +dox mice exhibited increased expression of hyperpolarization-activated cyclic nucleotide-gated potassium channel 4 (*Hcn4*) and downregulation of gap junction protein α 5 (*Gja5*) in atria (Figure 6E). HCN4 contributes to the native pacemaker current in the heart and is involved in the regulation of heart rhythm. Mutations in *HCN4* have been associated with sick sinus syndrome (45) and early-onset atrial fibrillation (46, 47). GJA5, a major gap junction protein in the atrial working myocardium, is responsible for intercellular propagation of the action potential. Somatic mutations in *GJA5* have been linked to atrial fibrillation (48) and *Gja5*-knockout mice exhibit conduction defects and a higher predisposition to atrial arrhythmias (49, 50). CUG960 +dox mice displayed downregulation of sodium voltage-gated channel α subunit 10 (*Scn10a*) in ventricles (Figure 6E). The *Scn10a* gene encodes the α subunit of voltage-gated sodium channel Nav1.8, previously shown to be expressed in intracardiac neurons and cardiomyocytes and linked in genetic association studies with QRS interval (51) and QT interval (52) duration. In both atria and ventricles, CUG960 +dox mice showed strong reduction in *Junctin* mRNA levels (Figure 6E). Junctin is a SR transmembrane protein that forms a quaternary protein complex with the ryanodine receptor, calsequestrin, and triadin and is involved in the regulation of calcium release from the SR. Downregulation of Junctin levels is observed in heart failure (53) and knockout mouse models show aberrant calcium homeostasis leading to arrhythmias (54).

CUG960 +dox mice exhibited alternative splicing defects in various genes involved in the regulation of the cardiac action potential (Figure 7E). The *SCN5A* gene encodes the major voltage-gated cardiac sodium channel Nav1.5, which is responsible for the influx of the Na+ ions (I_Na_) contributing to the depolarization phase of the action potential. The voltage-sensing transmembrane region of SCN5A located in domain I, segments S3 and S4, is encoded by 2 developmentally regulated, mutually exclusive exons 6A and 6B. While exon 6B is predominantly included in the adult heart, *SCN5A* mis-splicing resulting in inclusion of the fetal exon 6A is observed in DM1 (42). Previous studies have shown that inclusion of the fetal exon 6A alters the electrophysiological properties of the channel resulting in slower channel kinetics and slower recovery from inactivation (55). Forced expression of the fetal isoform in adult mouse heart resulted in cardiac conduction defects and arrhythmias (42, 56). CUG960 +dox mice exhibited a switch from predominant inclusion of exon 6B to partial inclusion of fetal exon 6A in both the atria and ventricles of the heart (Figure 7E). The voltage-gated potassium channel Kv4.3, encoded by the *KCND3* gene, effluxes potassium ions, generating the transient outward current (I_to1_) that contributes to the repolarization phase of the cardiac action potential. Kv channel interacting protein 2 (KChIP2), encoded by the *KCNIP2* gene, serves as an auxiliary protein, which interacts with and modulates the activity of the Kv potassium channels such as Kv4.2 and Kv4.3. As observed in DM1 (40, 42), CUG960 +dox mice display skipping of *Kcnd3* exon 6 and *Kcnip2* exon 3 (Figure 7E). The alternatively spliced region in *KCND3* encodes 19 amino acids at the C-terminal, including a threonine residue, which can be phosphorylated by PKC to regulate I_to_ (57), and the isoform lacking the exon shows faster inactivation, thereby contributing to lengthening of action potential duration (58). The KChIP2 isoform lacking exon 3 (KChIP2b) has been previously shown in in vitro studies to confer differential activation kinetics and recovery from inactivation for Kv4.3 channel gating (59).

We also observed alternative splicing changes in genes associated with the functions of calcium handling in the heart. RyR2, the predominant isoform of the ryanodine receptor family of ion channels expressed in the heart, regulates the flow of calcium ions out of the SR in cardiomyocytes. Abnormalities in RyR2 gating have been implicated in the development of atrial arrhythmias (60). In both the atria and the ventricles, CUG960 +dox mice exhibited skipping of exon 4 and 5 for *Ryr2* (Figure 7E). The exclusion of *RyR2* exon 4 was recently demonstrated in iPSC-derived DM1 cardiomyocytes (61). However, the functional significance of the region encoded by this exon is currently unknown. CaMKIIδ is a multifunctional Ser/Thr protein kinase involved in the regulation of calcium homeostasis by targeting various proteins involved in the calcium uptake and release such as the ryanodine receptors, phospholamban, and L-type calcium channels. The multiple *Camk2d* splice variants expressed in the heart can be distinguished based on the inclusion of exons 14, 15, and 16, which alter the cellular localization and function of the protein (62). CUG960 +dox mice exhibited decreased inclusion of exons 14 and 15 and an overall shift in *Camk2d* splicing pattern toward the δ_C_ isoform lacking exons 14, 15, and 16 (Figure 7E), which localizes to the cytoplasm and has been shown to phosphorylate RyR2 and phospholamban (63). Enhanced CaMKIIδ phosphorylation of RyR2 has been shown to promote atrial arrhythmias (64).

Consistent with the observed transcriptomic alterations, we identified calcium-handling defects in atrial cardiomyocytes isolated from CUG960 +dox mice in comparison with controls. Increased diastolic calcium release from RyR2 in the form of calcium waves coupled with increased sodium influx though NCX can lead to delayed-after-depolarizations (DADs). DADs large enough to reach the excitation threshold for an action potential can trigger ectopic beats that contribute to arrhythmogenesis (44, 65).

In conclusion, we developed a CUGexp RNA–expressing mouse model of DM1 that recapitulated various physiological, cellular, and molecular aspects of cardiac DM1 pathogenesis. Importantly, these phenotypes are reversible in response to cessation of transgene expression, underscoring the therapeutic potential of targeting CUGexp RNA for reversal of disease features. Molecular transcriptomic analysis revealed dysregulation of several genes associated with the functions of ion transport and regulation of cardiomyocyte action potential at the level of mRNA abundance and alternative splicing. Isolated atrial cardiomyocytes from repeat RNA–expressing mice exhibited calcium-handling defects, which could potentially contribute to the observed arrhythmogenic phenotypes. Future work will entail detailed mechanistic investigations into individual candidate genes for their contribution to DM1 cardiac pathology.

## Methods

### Transgenic mice.

TREDT960I mice were developed in an FVB background by pronuclear injection of linearized transgene using standard techniques as described (38). The TREDT960I transgene contains 960 interrupted CTG repeats in the context of a human genomic segment containing *DMPK* exons 11–15. The interrupted repeats were generated by ligating fragments containing 20 CTG repeats, which are separated by 5 nucleotide interruptions created by fusion of SalI and XhoI restriction sites. The repeats are stable for propagation in mice and bacteria. Southern blotting analysis confirmed that the number of repeats in the integrated transgene are stable across multiple generations (data not shown). The transgene is flanked by cHS4 insulator sequences on the 5′ and 3′ ends to prevent any effects associated with chromosomal integration sites (66). The TREDT960I mice are available from The Jackson Laboratory (stock no. 032050). MHC-rtTA transgenic mice (FVB/N-Tg(Myh6-rtTA)1Jam) expressing a codon-optimized variant of rtTA specifically in the heart were commercially obtained (RRID: MMRRC_010478) (39). Mice that were homozygous for TREDT960I and MHCrtTA transgenes were mated to mice homozygous for the TREDT960I transgene to obtain F1 progeny of bitransgenic mice homozygous for the TREDT960I transgene and hemizygous for MHCrtTA transgene, which were used for experiments. Mice hemizygous for MHCrtTA transgene that were used as controls were obtained by mating MHCrtTA hemizygous mice to wild type FVB mice. The study consisted of both male and female animals and is not gender specific. Mice were provided with dox-containing chow (2 g dox/kg chow, Bioserv) either beginning at PN1 through nursing dams or at 2 months or 6 months of age. Genomic DNA was isolated from tail clips using DirectPCR lysis reagent (Viagen Biotech) and evaluated by PCR using transgene-specific primers for genotypes. Primer sequences are provided in Supplemental Table 2.

### RT-qPCR and RNA splicing.

Total RNA was isolated from atrial and ventricular heart tissues using TRIzol reagent (15596-018; Invitrogen). cDNA was prepared from 1 μg of DNase treated RNA (4368813; Thermo Fisher Scientific). Primers targeting human *DMPK* exons 12–14 were used to assay transgene expression by real-time qPCR. Mouse *Rpl4* was used as internal control for normalization. PCR reactions were carried out on an Applied Biosystems 7500 Fast Real-Time PCR System using PowerUp SYBR-Green PCR master mix (Thermo Fisher Scientific). Relative expression levels were determined by the 2^-ΔΔCt^ method. For analysis of alternative splicing events, primers annealing to flanking constitutive exons were designed. PCR products were resolved on a 5% polyacrylamide gel. Ethidium bromide stained RT-PCR bands were analyzed using Kodak Gel Logic 2000 and Carestream software. Percent spliced in (PSI) values were calculated using densitometry according to the equation: PSI = 100 X [Inclusion band/(Inclusion band + Skipping band)]. Primer sequences are provided in Supplemental Table 2 and Supplemental Table 3. See complete unedited blots in the supplemental material.

### Immunoblotting.

Tissue protein extracts were prepared from atrial and ventricular heart tissues by homogenization followed by sonication in RIPA lysis buffer containing 1× Halt protease and phosphatase inhibitor cocktail (Thermo Fisher Scientific). Cellular debris was cleared by centrifugation (16,000*g* for 30 minutes at 4°C). Protein lysates (20–25 μg) were separated on 12% Tris-glycine SDS-PAGE gels and transferred to nitrocellulose membranes for Western blot analysis. Total protein was visualized by Ponceau S staining and membranes were incubated in anti-CELF1 (3B1, catalog 05-621, EMD Millipore, 1:500 dilution) or anti-GAPDH (14C10, catalog 2118, Cell Signaling Technology, 1:200,000 dilution) primary antibodies overnight at 4°C. Membranes were washed 3 times in PBS (0.1% Tween-20, Sigma) and incubated for 2 hours in HRP-conjugated goat anti–rabbit or goat anti-mouse (1:10,000, Jackson Immunoresearch) antibodies. After washing 3 times in PBST (0.1% Tween-20), blots were imaged on a ChemiDoc XRS+ Imaging system (Bio-Rad). See complete unedited blots in the supplemental material.

### Echocardiography.

Echocardiographic examination of in vivo cardiac function and morphology was performed using a Vevo 2100 ultrasound machine equipped with a 40 MHz transducer-MS550S (Visualsonics). For imaging, mice were anesthetized with 1.5% isoflurane and body temperature was maintained at 37°C. M-mode images were acquired in the short-axis position at the level of papillary muscles for each animal. Three M-mode tracings were analyzed per animal using the Visualsonics VevoLab analysis package and values were averaged.

### ECG.

ECG data were obtained using a multichannel amplifier followed by conversion to digital signals for analysis. Surface ECG recordings were measured from 3 leads using a Rodent Surgical Monitor+ platform (Indus Instruments) and data were acquired using PowerLab 8/35 (AD Instruments). Mice were anesthetized using 2% isoflurane for data collection. Body temperature was monitored using a rectal probe. Data were collected for 1.5–2 minutes per mouse and ECG intervals were analyzed using LabChart software (AD Instruments). The QTc interval was calculated using Bazett’s formula (67).

### Intracardiac programmed electrical stimulation.

Programmed intracardiac stimulation was performed to evaluate inducibility of atrial arrhythmias as previously described (68). Briefly, an incision was made to the right of the midline near the clavicle to access the right jugular vein for insertion of a 1.1F octapolar catheter (EPR-800; Millar Instruments) into the right atrium and ventricle. Atrial arrhythmias were induced using a burst pacing protocol where a 2-second burst with a cycle length of 40 ms was applied followed by successive bursts each with 2 ms decrements down to a cycle length of 10 ms. Pacing protocols were performed in triplicate and mice were considered positive for arrhythmia inducibility if pacing evoked arrhythmias lasting 1 second or longer, at least 2 out of 3 times. The incidence of inducible atrial arrhythmia was calculated as the percentage of arrhythmia-positive mice divided by the total number of mice studied.

### FISH-IF.

Combined FISH-IF was performed on frozen cardiac sections (7 μm) using a modified protocol as previously described (8). Sections were fixed 30 minutes with 4% PFA/1× PBS at room temperature, washed 5 times with 1× PBS for 2 minutes each followed by permeabilization in 2% prechilled acetone/1× PBS for 10 minutes. Sections were incubated with 30% formamide/2× saline sodium citrate (SSC) for 30 minutes, hybridized with Tye-563-labeled (CAG5 LNA probes (0.5 ng/μl, Exiqon-Qiagen) for 4.5 hours at 42°C in hybridization buffer (30% formamide, 2× SSC, 0.02% BSA, 66 μg/mL yeast tRNA, 2 mM Vanadyl ribonucleoside complex), and washed for 30 minutes in 30% formamide/2× SSC at 42°C. Sections were washed in 1× SSC for 30 minutes at room temperature followed by incubation in mouse monoclonal anti-MBNL1 (3A4, catalog sc-47740, Santa Cruz Biotechnology, 1:25 dilution) and anti-MBNL2 (3B4, catalog sc-136167, Santa Cruz Biotechnology, 1:25 dilution) antibodies overnight at 4°C. Sections were washed 5 times in 1× PBS for 2 minutes, incubated in Alexa 488–labeled goat anti-mouse secondary antibody (1:1000, Invitrogen), washed 3 times with 1× PBS for 5 minutes each, incubated with DAPI (0.5 μg/mL), washed 5 times with 1× PBS for 2 minutes each, and mounted in Shandon Immu-Mount (Thermo Fisher Scientific). Images were acquired using a DeltaVision Elite (GE Healthcare) and processed using SoftWoRx software (GE Healthcare).

### RNA-Seq analysis.

RNA was isolated from atria and ventricles using the RNeasy Fibrous Tissue Mini Kit (QIAGEN). The Genomic and RNA Profiling Core first conducted sample quality checks using the NanoDrop spectrophotometer and Agilent Bioanalyzer 2100. The samples passing the following criteria were used for RNA-Seq: RNA integrity number (RIN) ≥ 7.0, A260nm/A280nm ≥ 1.9, A260nm/A230nm ≥ 1.2, and r28S/16S ≥ 1.5. The Illumina TruSeq Stranded mRNA library preparation protocol was then used to generate cDNA libraries starting with 250 ng of total RNA. ERCC RNA Spike-In Controls were added to each sample according to the manufacturer’s protocol. The resulting libraries were quantitated using the NanoDrop spectrophotometer and fragment size assessed with the Agilent Bioanalyzer. The pooled libraries were loaded onto a NovaSeq 6000 S4 flow cell and sequenced to a depth of approximately 150 million read pairs/sample. A paired-end 150 cycle run was used to sequence the flow cell on a NovaSeq 6000 sequencing system. The data set has been deposited in the National Center for Biotechnology Information/Gene Expression Omnibus under accession number GSE164825. The raw fastq files were first quality checked using FastQC (version 0.11.8) software (http://www.bioinformatics.bbsrc.ac.uk/projects/fastqc/). Fastq files were aligned to mm10 mouse reference genome (GRCm38.39) and per-gene counts quantified by RSEM (69) (version 1.3.1) based on the gene annotation Mus_musculus.GRCm38.89.chr.gtf. Differential gene expression values were obtained using DESeq2 (70) (version 1.22.2). Gene expression differences were considered significant if passing the following criteria: adjusted *P* value < 0.05, log_2_(fold change) ≥ 1.5. For splicing analysis, reads were aligned to mm10 mouse reference genome (GRCm38.89) using STAR (71) (version 2.6.1a). Mapping percentage and sample details are provided in Supplemental Table 1. Based on the bam files generated from the alignment, splicing was quantified using rMATS (72) (version 4.0.2), which annotated and statistically analyzed 5 different kinds of splicing events (AS 5′SS, AS 3′SS, CE, MXE, and IR) using Mus_musculus.GRCm38.89.chr.gtf gene annotation. Splicing events were considered significant if passing the following criteria: reads ≥ 20, FDR ≤ 0.05, and ΔPSI ≥ 0.15. Gene ontology analysis was performed using DAVID (version 6.8). Enriched categories with –log_10_(*P* value) ≥ 1.5 (*P* value ≤ 0.032) were considered significant.

### Cellular Ca^2+^ imaging.

Atrial myocytes were isolated from adult mouse hearts using a retrograde Langendorff perfusion protocol as previously described (73). Cardiomyocytes that failed to follow pacing or did not have regular striations were not included in the study. Cells that were exhibiting pacemaker-like qualities with regular spontaneous depolarization or contractions were also not included in the study. Myocytes were incubated with 2 μM Fluo-4-AM (Invitrogen) and imaging was performed in line scan mode on a Zeiss LSM 880 confocal microscope. Fluo-4–loaded myocytes were placed in a chamber equipped with parallel platinum electrodes and steady-state Ca^2+^ transients (CaTs) were induced by 1Hz pacing (5 ms, 10V). Pacing was then stopped for 20 seconds and Ca^2+^ sparks were counted. For measurements of total SR Ca^2+^ content (SR load), perfusate was switched to calcium-free Tyrode solution to block L-type calcium channel and prevent any new cellular entry of calcium. SR load was determined by application of 10 mM caffeine after pacing. All transient measurements were normalized to baseline fluorescence (F0) as performed in previous studies (73). The incidence of Ca^2+^ waves was determined by calculating the number of cells displaying one or more noninduced CaTs (in the absence of pacing) divided by the total number of cells. SERCA activity was calculated as the difference between the decay rate of pacing-induced CaT (which reflects Ca^2+^ extrusion by combined activities of NCX and SERCA) and the decay rate of caffeine-induced CaT (which reflects Ca^2+^ extrusion by NCX only) as previously described (74). Data were analyzed using Clampfit and ImageJ (NIH) with a SparkMaster plugin.

### Statistics.

All quantitative experiments have at least 3 independent biological replicates. Results are presented as mean ± SD. Statistical analyses for data sets were carried out using Prism software (version 8.0, GraphPad) and methods used are specified in figure legends. Statistical tests included 1-way ANOVA followed by Tukey’s test for multiple comparisons; 2-way ANOVA followed by Tukey’s test for multiple comparisons; Fisher’s exact test; 2-tailed t test; and Kruskal-Wallis 1-way ANOVA followed by Dunn’s test for multiple comparisons. Calcium imaging data were analyzed using the Generalized Estimating Equation function (SPSS version 25.0.0.0) where mouse number was set as the subject variable, cell number as within subject variable, and each respective calcium measurement as the dependent variable. A *P* value of less than 0.05 was considered statistically significant.

### Study approval.

All mouse experiments were carried out in accordance with the *Guide for the Care and Use of Laboratory Animals* (National Academies Press, 2011) and approved by the Baylor College of Medicine Institutional Animal Care and Use Committee.

## Author contributions

ANR designed the study, performed experiments, analyzed the data, and wrote the manuscript. HMC performed intracardiac pacing experiments, and TAW performed calcium imaging studies under the supervision of XHTW. ZX and XG performed computational analysis of sequencing reads from RNA-Seq data. TAC supervised and designed the study, analyzed the data, and wrote the manuscript. All authors contributed to the interpretation of results and production of the final manuscript.

## Supplementary Material

Supplemental data

## Version 1 01/26/2021

In-Press Preview

## Version 2 03/08/2021

Electronic publication
