# Supplementary material for: Reversible cardiac disease features in an inducible CUG repeat RNA–expressing mouse model of myotonic dystrophy
Source: JCI Insight. 2021 Mar 8;6(5):e143465. doi: 10.1172/jci.insight.143465 (PMC8021116; doi:10.1172/jci.insight.143465)
Supplement: Supplemental data [file jciinsight-6-143465-s255.pdf]

Supplemental figure 1

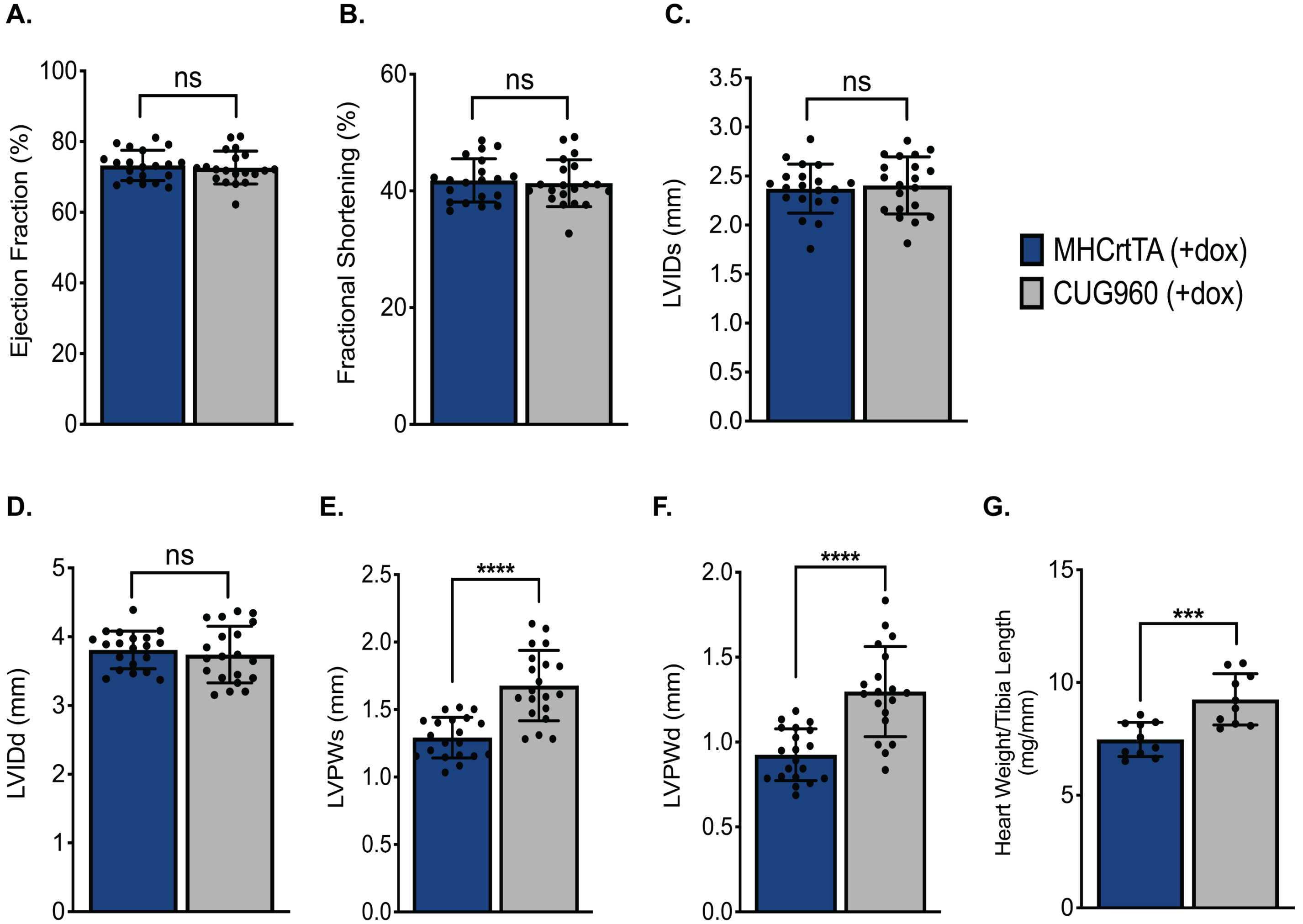

Supplemental Figure 1. CUG960 mice display increased heart size and ventricular wall thickening. M-mode echocardiography was performed on CUG960 +dox mice induced to express repeat RNA beginning at PN1 for 2 months to evaluate A) ejection fraction B) fractional shortening C) left ventricular internal diameter systole D) left ventricular internal diameter diastole E) left ventricular posterior wall thickness systole F) left ventricular posterior wall thickness diastole in comparison to MHCrtTA +dox control mice. n=20 animals per group. G) Heart weight was normalized to tibia length. For MHCrtTA, n=9 animals and for CUG960, n=11 animals. Data represent the mean ± SD and were analyzed using two-tailed t-test. *p<0.05, **p<0.01, ***p<0.001, ****p<0.0001.

Supplemental figure 2

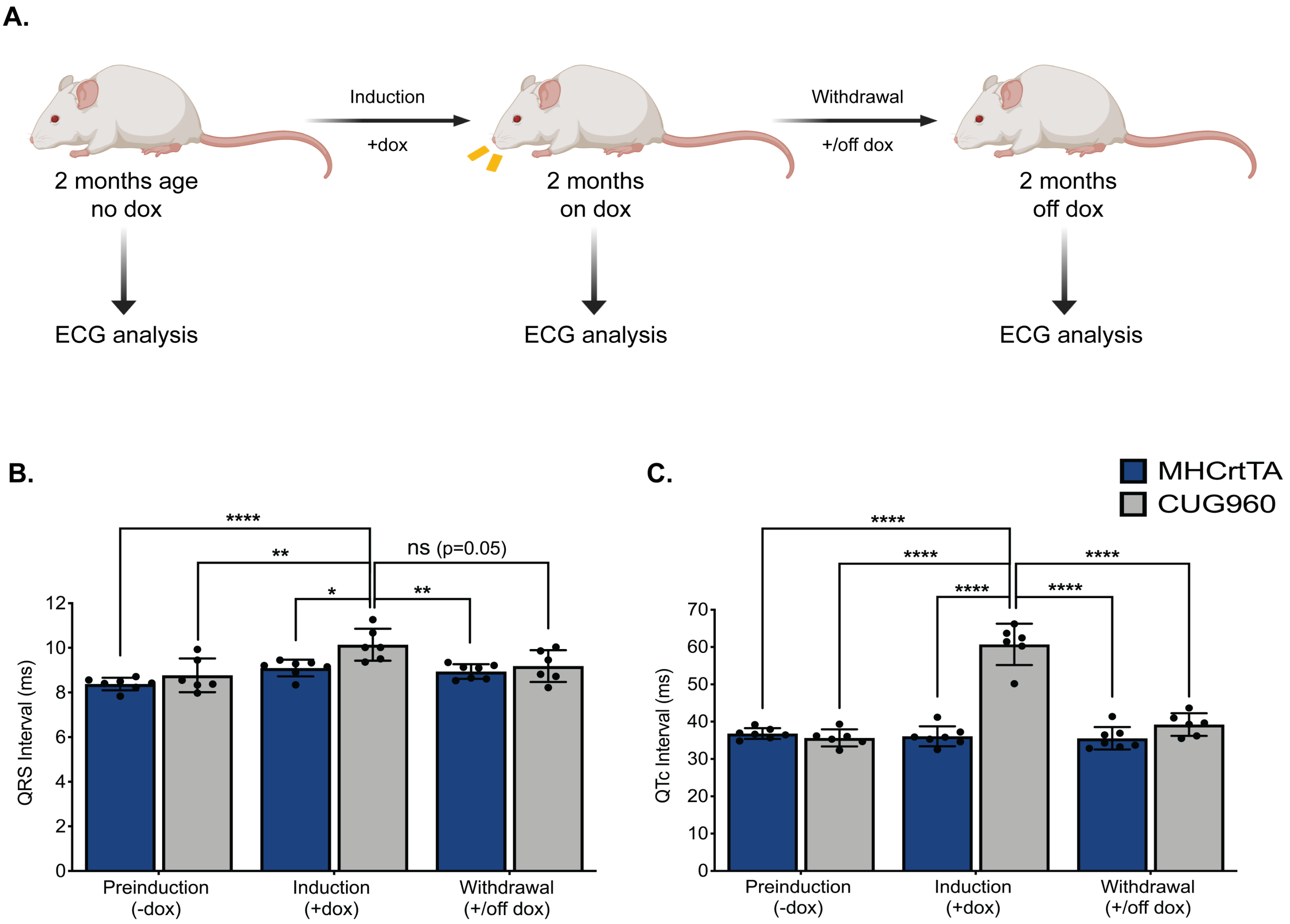

Supplemental Figure 2. CUG960 mice induced to express CUGexp RNA as adults show conduction abnormalities that are reversible in response to diminished repeat RNA expression. A) Surface ECG recordings were obtained to evaluate conduction intervals in CUG960 and MHCrtTA control mice before CUGexp RNA induction at 2 months of age, then after induction on dox chow for 2 months and then again after a switch to regular chow for 2 months: B) QRS interval and C) corrected QT (QTc) interval. For MHCrtTA, n=7 animals and for CUG960, n=6 animals. Data represent the mean ± SD and were analyzed using two-way ANOVA followed by Tukey's test for multiple comparisons. *p<0.05, **p<0.01, ***p<0.001, ****p<0.0001.

Supplemental figure 3

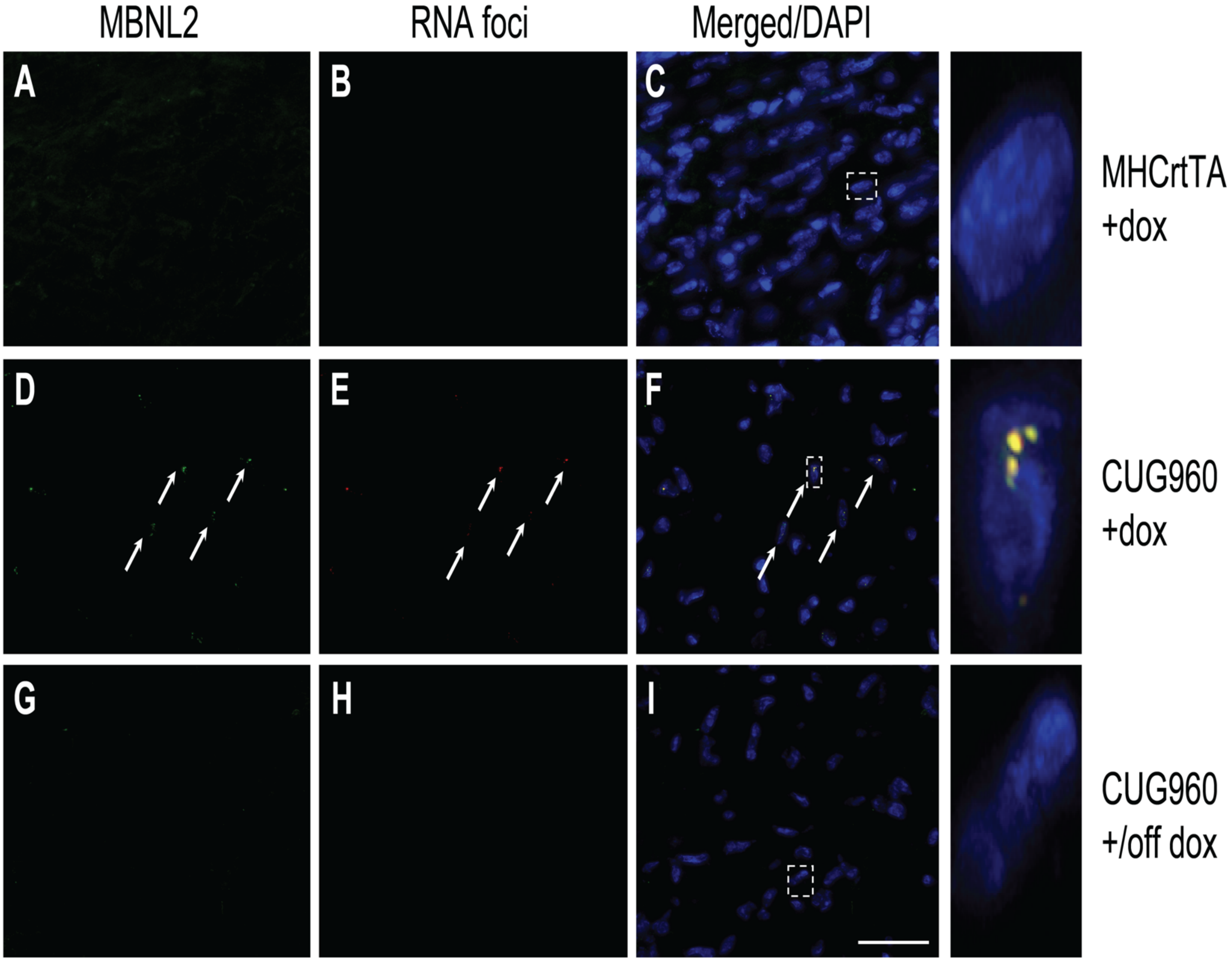

Supplemental Figure 3. CUG960 mice display reversible nuclear RNA foci formation and MBNL2 colocalization. RNA FISH with probe targeting CUG repeat RNA combined with immunofluorescence for MBNL2 in the ventricles of CUG960 mice in response to dox induction (D-F) and withdrawal (G-I) in comparison to MHCrtTA +dox control mice (A-C). Arrows indicate RNA foci colocalized with MBNL2 staining. Scale bar: 25 microns.

Supplemental figure 4

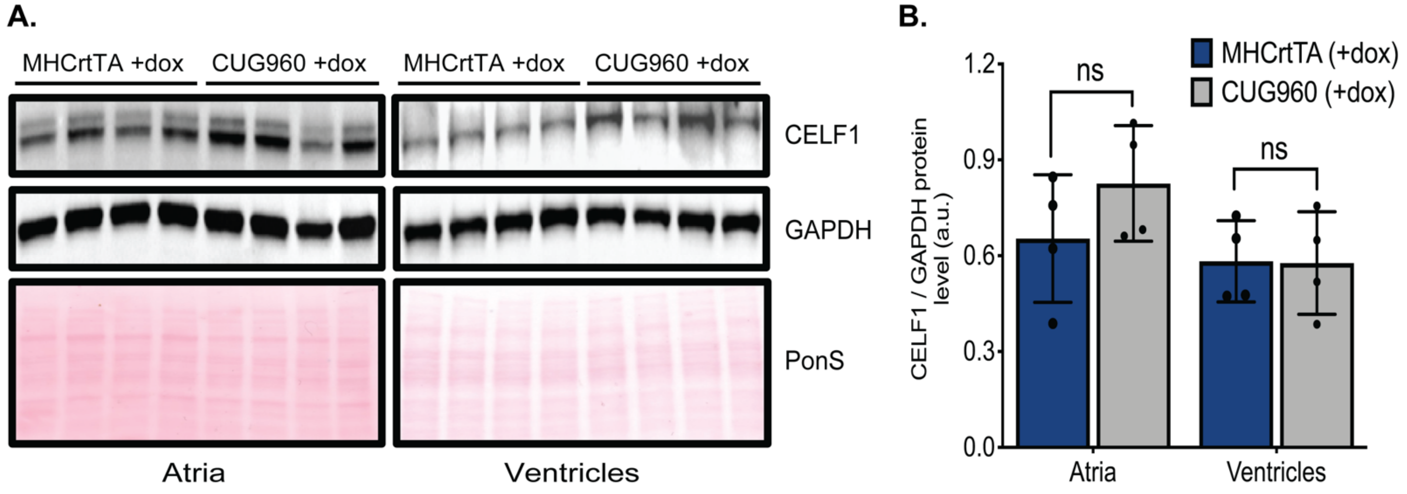

Supplemental Figure 4. CUG960 mice do not show elevation of CELF1 protein levels in comparison to controls. A) Western blotting and B) quantification for CELF1 protein levels in atria and ventricles from CUG960 +dox and MHCrtTA +dox control mice. n=4 animals per group. Data represent the mean ± SD and were analyzed using two-tailed t-test.

Supplemental figure 5

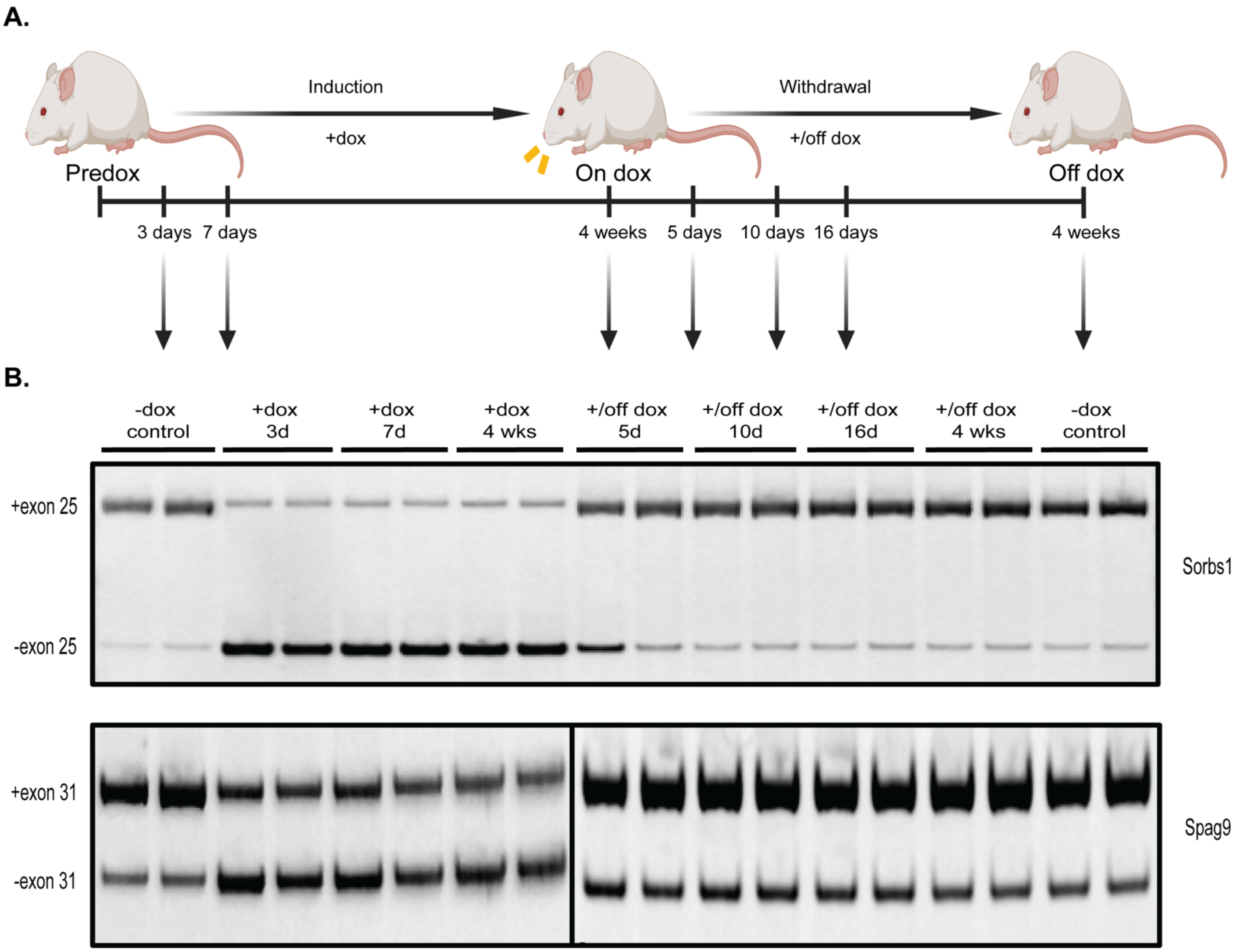

Supplemental Figure 5. Splicing time course experiment. A) Ventricular RNA was isolated from CUG960 mice given dox chow starting at 6 months of age for 3 days, 1 week or 4 weeks or taken off dox food for 5 days, 10 days, 16 days or 4 weeks after 4 weeks of induction. B) Representative RT-PCRs showing inducibility of splicing defects for *Sorbs1* exon 25 and *Spag9* exon 31 in ventricles of CUG960 +dox mice as early as 3 days post induction. Splicing defects are nearly completely rescued in CUG960 mice as early as 5 days following dox withdrawal. n=2 animals per group.

Supplemental figure 6

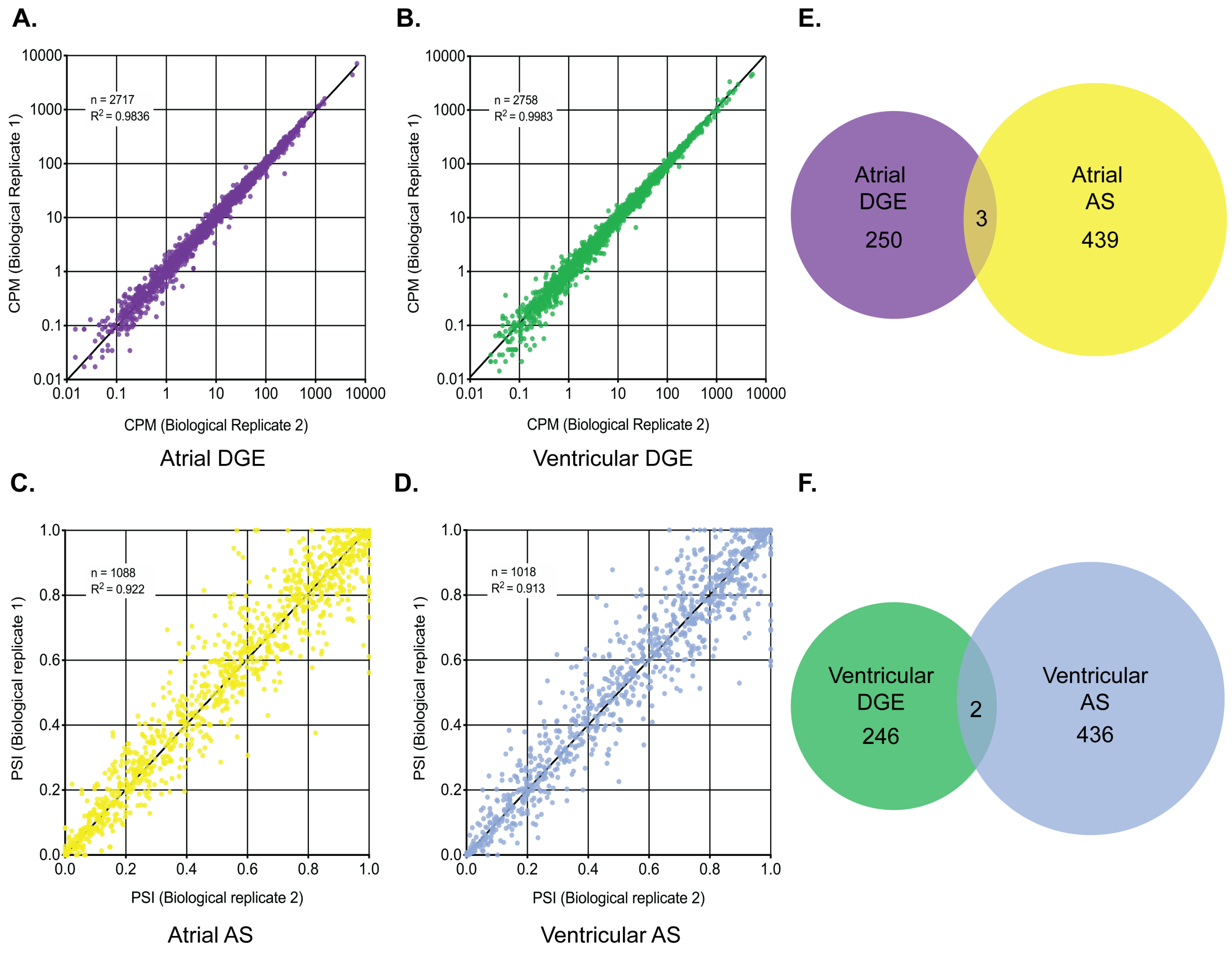

Supplemental Figure 6. Reproducibility and overlap observed for DGE and AS (cassette exon) changes in atria and ventricles. Biological replicate RNA-seq data sets (CUG960 +dox, PN1 induction) for A) atria and B) ventricles, analyzed for reproducibility in gene expression. CPM: counts per million reads mapped, n = number of genes. Biological replicate RNA-seq data sets (CUG960 +dox, PN1 induction) for C) atria and D) ventricles, analyzed for reproducibility in alternative splicing. PSI: Percent Spliced In, n = number of splicing events. E) Overlap observed between genes showing expression level and alternative splicing changes in atria. F) Overlap observed between genes showing expression level and alternative splicing changes in ventricles.

Supplemental figure 7

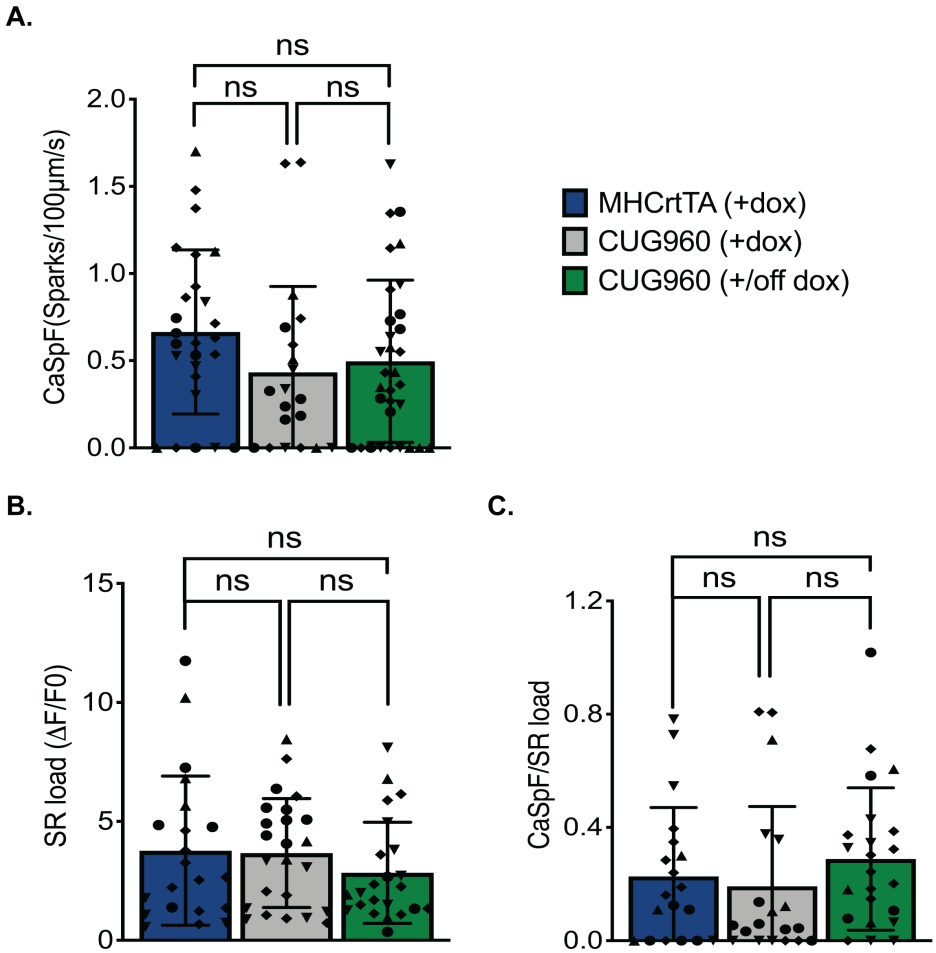

Supplemental Figure 7. CUG960 +dox mice do not show changes in calcium spark frequency or SR load. A) Spontaneous Ca^{2+} spark frequency (CaSF) B) Sarcoplasmic reticulum (SR) Ca^{2+} load C) CaSF normalized to caffeine-induced SR Ca^{2+} load in CUG960 +dox mice in comparison to MHCrtTA +dox controls and CUG960 +/off dox mice. n=4 animals per group and data points represent individual cardiomyocytes. Within each group, all cardiomyocytes from each mouse are depicted with distinct symbols. Data represent the mean ± SD and were analyzed using the Generalized Estimating Equation function in SPSS.

Supplemental Table 1. RNA-sequencing sample information

Sample	Genotype	Tissue	Sex	Uniquely Mapped Reads	% Reads Uniquely Mapped to Genome
V327A	MHCrtTA +dox	Atria	Female	132689172	85.25%
V333A	MHCrtTA +dox	Atria	Female	146860729	84.83%
V334A	MHCrtTA +dox	Atria	Female	134747794	85.01%
V327V	MHCrtTA +dox	Ventricle	Female	121098848	74.85%
V333V	MHCrtTA +dox	Ventricle	Female	116302012	75.21%
V334V	MHCrtTA +dox	Ventricle	Female	120203793	80.64%
V119A	CUG960 +dox	Atria	Female	117564708	83.62%
V120A	CUG960 +dox	Atria	Female	131946839	83.59%
V123A	CUG960 +dox	Atria	Female	113888495	84.37%
V119V	CUG960 +dox	Ventricle	Female	148290213	81.84%
V120V	CUG960 +dox	Ventricle	Female	143164420	80.47%
V123V	CUG960 +dox	Ventricle	Female	131732206	76.92%

Supplemental Table 2. Primer sequences used in genotyping and RT-qPCR experiments.

Type	Transgene / Target Gene	Assay	Primer	Sequence (5' → 3')
**Genotyping**	TREDT960I exon 15	PCR	Forward	GTTCGCCGTTGTTCTGT
	TREDT960I exon 15	PCR	Reverse	TCGGAGCGGTTGTGAACT
	MHCrtTA	PCR	Forward	CTGGGTTGCGTGTTGGAAGATC
	MHCrtTA	PCR	Reverse	GTGGGAGATCGAGCAGGCCCTCG
**Gene**	TREDT960I exon 12-14	RT-qPCR	Forward	CAGGAGCGGATGGAGTTG
**expression**	TREDT960I exon 12-14	RT-qPCR	Reverse	GCAGGGAGCAGCAGGTG
	*Hcn4*_mouse	RT-qPCR	Forward	CTATGAACACCGCTACCAAGG
	*Hcn4*_mouse	RT-qPCR	Reverse	CAAAGTTGGGATCTGCGTTG
	*Gja5*_mouse	RT-qPCR	Forward	CCTCGGTCTCCTACTCTTGG
	*Gja5*_mouse	RT-qPCR	Reverse	CATGCGGAAAATGAACAGGAC
	*Scn10a*_mouse	RT-qPCR	Forward	TGTATGCAGCTGTCGATTCTC
	*Scn10a*_mouse	RT-qPCR	Reverse	ATGACCCCGACAAAGAGATTC
	*Junctin*_mouse	RT-qPCR	Forward	GGCGATGGAGACTTTGATGT
	*Junctin*_mouse	RT-qPCR	Reverse	CAGCTCTTCTTTGATGGGTTC
	*Rpl4*_mouse	RT-qPCR	Forward	CGCTGGTGGTTGAAGATAAGG
	*Rpl4*_mouse	RT-qPCR	Reverse	CGGTTTCTCATTTTGCCCTTG

Supplemental Table 3. Primer sequences used in RT-PCR experiments.

Type	Target Gene	Assay	Primer	Sequence (5' → 3')
**Alternative**	*Sorbs1*_exon 25_mouse	RT-PCR	Forward	CCAGCTGATTACTTGGAGTCCACAGAAG
**splicing**	*Sorbs1*_exon 25_mouse	RT-PCR	Reverse	GTTCACCTTCATACCAGTTCTGGTCAATC
	*Spag9*_exon 31_mouse	RT-PCR	Forward	GGACTGGAAATGGTGTCATTATCTCCAT
	*Spag9*_exon 31_mouse	RT-PCR	Reverse	GGGACTGCCACAAAGAATTTCACAG
	*Tnnt2*_exon 4,5_mouse	RT-PCR	Forward	GTACGAGGAGGAACAGGAAG
	*Tnnt2*_exon 4,5_mouse	RT-PCR	Reverse	CCAGCCTCCTCCTCCTCC
	Mef2d _β exon_mouse	RT-PCR	Forward	AGGGAGGCAAAGGGTTAATG
	Mef2d _β exon_mouse	RT-PCR	Reverse	CCCTGGCTGAGTAAACTTGG
	*Scn5a*_exon 6A_mouse	RT-PCR	Forward	GTCGGCTCTTCGAACTTTCA
	*Scn5a*_exon 6B_mouse	RT-PCR	Forward	CGGGCCCTGAAAACTATATC
	*Scn5a*_exon 7_mouse	RT-PCR	Reverse	CCAATGAGGGCAAAGACACT
	*Ryr2*_exon 4,5_mouse	RT-PCR	Forward	CGGACCTGTCTATCTGCACCTTTGT
	*Ryr2*_exon 4,5_mouse	RT-PCR	Reverse	CATACCACTGTAGGAATGGCGTAGCA
	*Camk2d*_exon 14, 15, 16_mouse	RT-PCR	Forward	TGAAGAAACCAGATGGGGTAA
	*Camk2d*_exon 14, 15, 16_mouse	RT-PCR	Reverse	CCTCAAAGTCCCCATTGTTG
	*Kcnd3*_exon 6_mouse	RT-PCR	Forward	GGCAAGACCACCTCACTCAT
	*Kcnd3*_exon 6_mouse	RT-PCR	Reverse	TGGCTGGACAGAGAAGGACT
	*Kcnip2*_exon 3_mouse	RT-PCR	Forward	CCCTGCCCTCAGTCAGTGAAA
	*Kcnip2*_exon 3_mouse	RT-PCR	Reverse	TGCGTGTGAACTTGGTTTGTTCC
